# Exploring miR-144-3p Expression and its Diagnostic Value in Peri-Implantitis

**DOI:** 10.3290/j.ohpd.c_2828

**Published:** 2026-09-29

**Authors:** Gaoming Yan, Haiping Song, Huan Lou

**Affiliations:** a Gaoming Yan Attending Physician, Department of Dentistry, Tangshan Hongci Hospital, Tangshan, China. Contributed substantially to conception and design, acquisition of data, analysis and interpretation of data, drafted the manuscript, read and approved the final manuscript.; b Haiping Song Attending Physician, Chengdu Qingyang Rongcheng Sansan Dental Clinic, Chengdu, China. Contributed substantially to conception and design, analysis and interpretation of data, drafted the manuscript, read and approved the final manuscript.; c Huan Lou Attending Physician, Periodontology Department, Ningbo Stomatological Hospital, Ningbo, China. Final revision of the manuscript, proofreading, communications, project supervision. Gaoming Yan and Haiping Song contributed equally to the study.

**Keywords:** biomarker, diagnosis, inflammation, miR-144-3p, peri-implantitis.

## Abstract

**Purpose:**

Micro-RNA (miRNA) is an emerging biomarker for periodontal disease. To determine its diagnostic value in peri-implantitis (PI) and its relationship with inflammatory factors, this study focused on miR-144-3p, potentially providing a much-needed new biomarker for PI.

**Materials and Methods:**

A total of 98 healthy subjects and 114 PI patients were included in this study. Clinical parameters such as plaque index (PLI), sulcus bleeding index (SBI), probing depth (PD), full-mouth plaque scores (FMPS), clinical attachment level (CAL), and bleeding on probing/suppuration were recorded and compared. The quantification of miR-144-3p was performed using RT-qPCR. The ROC curve assessed the diagnostic value. The chi-squared test was utilized to analyze associations between the clinicopathological characteristics of PI and miR-144-3p level. Risk factors for PI were analyzed using logistic regression. HGFs were treated with LPS as the PI model. miR-144-3p was overexpressed or inhibited by transfection into the PI model. Then, cell viability and inflammatory factors were detected in the PI model.

**Results:**

Compared to controls, PI patients had statistically significantly higher PLI, SBI, PD, FMPS, CAL, and a higher rate of bleeding on probing/suppuration. The miR-144-3p level was increased and had high diagnostic value in PI. The presence of miR-144-3p was a risk factor for PI. In addition, the miR-144-3p level was closely related to clinical indicators in PI. In the PI model, overexpression of miR-144-3p reduced proliferation and increased inflammatory factor levels, while inhibition of miR-144-3p increased proliferation and reduced inflammatory factor levels.

**Conclusion:**

miR-144-3p correlated with clinical parameters such as PLI, SBI, PD, FMPS, CAL, and bleeding on probing/suppuration and participated in PI by regulating cell viability and inflammatory factors, supporting its potential as a clinically relevant biomarker for PI.

Dental implants have become an indispensable and highly successful restorative treatment method.^31^ However, they may be susceptible to peri-implantitis (PI), which is a plaque-induced inflammatory disease that leads to pathological bone resorption around dental implants.^23^ The accumulation of dental plaque is a major driver of PI, capable of inducing inflammatory tissue lesions and consequent alveolar bone loss.^38^ Beyond plaque accumulation, emerging risk factors such as inadequate soft tissue dimensions, prosthesis design, and surgical positioning may further increase susceptibility to PI.^22^ Currently, the success rate of dental implants is more than 95%, but there are occasional failures, sometimes due to PI.^2^ The peri-implant soft tissue serves to seal the implant interface, thereby establishing an epithelial barrier that is crucial for maintaining homeostasis and protecting against bacterial invasion.^8^ However, if integration between the soft tissue and the implant is poor or lacking, Gram-negative anaerobic bacteria invading the peri-implantitis area will activate the inflammatory response and produce inflammasomes, thereby leading to PI.^3^

Recent epidemiological data show that severe periodontitis increases the risk of PI.^26^ The failure of dental implants can lead to an increase in the patient’s economic burden and a decline in their quality of life. The diagnosis of PI traditionally relies on clinical probing and bleeding assessment, but the sensitivity of these parameters is limited. In order to clearly distinguish from peri-implant mucositis, radiological testing has been introduced.^24,29^ A comprehensive approach integrating clinical, radiographic, and biomarker assessments is needed for long-term peri‑implant stability.^27^ Moreover, compared with periodontitis, there is currently no established and predictable concept of PI treatment, and the disease has become a major burden in implant dentistry.^5,13^ Therefore, there is a pressing need to identify a reliable biomarker for the early diagnosis and risk assessment of PI.

MicroRNA (miRNA) affects periodontal morphology, function, and gene expression during periodontal disease,^18,19^ suggesting that miRNA is an emerging biomarker for periodontal disease. However, there are relatively few studies on miRNA in PI. This study focused on miR-144-3p, which is linked with various diseases, such as myocardial infarction,^39^ depression,^34^ and diabetes.^40^ Notably, miR-144-3p is closely related to inflammation. In *Mycobacterium* abscess infection, there is a strong correlation between miR-144-3p and pro-inflammatory cytokines/chemokines.^14^ miR-144-3p is associated with the proliferation, apoptosis, and inflammation of fibroblasts in osteoarthritis.^37^ PI is an inflammatory disease. Therefore, we speculate that miR-144-3p might participate in PI. Studies have shown that PI is linked with abnormal osteogenic differentiation.^10^ There are also findings which demonstrate that miR-144-3p acts as a critical negative regulator of osteogenic differentiation in mouse mesenchymal stem cells (MSCs), suggesting its potential as a therapeutic target for bone regeneration disorders.^12^ In addition, in mandibular bone marrow MSCs, miR-144-3p is also related to osteogenic differentiation.^33^ The above evidence also confirms that miR-144-3p has the potential to become a biomarker of PI.

We hypothesized that miR-144-3p may be significantly upregulated in PI, positively correlate with clinical parameters, and have high diagnostic accuracy for PI. The main objective was to determine the diagnostic value of miR-144-3p in PI and its relationship with inflammatory factors, providing a new biomarker for PI.

## MATERIALS AND METHODS

### Patients and Specimens

From March 2021 to April 2024, 212 subjects from Tangshan Hongci Hospital were included. Among them were 114 subjects in the PI group and 98 subjects in the control group. The inclusion criteria were PI according to the criteria established at the 2017 Symposium on the Classification of Periodontal and peri-implant diseases and disorders.^4^ The exclusion criteria were as follows: pregnancy or breastfeeding; suffering from a systemic disease; other oral infections or lesions.

All procedures performed in studies involving human participants were in accordance with the 1964 Helsinki Declaration. This study was approved by the Ethics Committee of Tangshan Hongci Hospital, and all patients signed the informed consent form. In the early morning, the subjects had been fasting and saliva samples were collected.

### Peri-Implant Parameters

To evaluate the conditions around the implants, the clinical parameters of dental implant patients were recorded utilizing a manual periodontal probe (PC-PUNC 15 Hu-Friedy; Chicago, IL, USA). Plaque index (PLI) was recorded, with a score of 0-3 for each tooth surface based on plaque thickness. Sulcus bleeding index (SBI) was recorded as a measure of bleeding on probing. Probing depth (PD) was measured in millimeters from the peri-implant margin to the base of the pocket. Full-mouth plaque score (FMPS) was recorded as the percentage of implant surfaces with visible plaque (presence/absence at each site). Clinical attachment level (CAL) was measured from the implant shoulder to the pocket base. Bleeding and/or suppuration on probing was recorded as present if either occurred within 30 s after probing.

### Cell Culture

Human gingival fibroblasts (HGFs) (Innoprot; Bizkaia, Spain) were cultured in DMEM (HyClone; Logan, UT, USA) containing 10% FBS and 1% penicillin and streptomycin solution. Experiments were conducted using cells of the 3rd to 6th generations. Then, 1 μg/ml *P. gingivalis* LPS (Invivogen; San Diego, CA, USA) was used to treat HGFs for different time (0, 4, 8, 12 h). Finally, HGFs treated with 1 μg/ml LPS at 12 h were selected as the PI model.

### Cell Transfection

To overexpress and inhibit miR-144-3p, miR-144-3p mimic or miR-144-3p inhibitor was transfected into the PI model. All plasmids were purchased from GenePharma (Shanghai, China).

### RT-qPCR

RNA from saliva was extracted using an miRNeasy Serum/Plasma Advanced Kit (Qiagen; Manchester, UK). cDNA was obtained by reverse transcription of RNA using the miRCURY LNA Universal Reverse Transcription Kit (Qiagen). miR-144-3p expression was quantified utilizing SYBR qPCR Mix and RT-qPCR system. Then, miR-144-3p expression was normalized to U6 and calculated by 2-ΔΔCT.

### Cell Counting Kit-8 (CCK-8) Assay

HGFs were resuspended at a density of 2 × 10^3^ cells/ml. A 100-μl aliquot of the cell suspension was seeded into each well of a 96-well plate. After 24 h of culture, 10 μl of CCK-8 solution was added to each well, followed by incubation for 1 h. The absorbance at 450 nm was then detected.

### ELISA

The concentrations of serum TNF-α and IL-1β were measured utilizing an ELISA kit (Shenzhen Xinbosheng Biological; Shenzhen, China). Standards and 5-fold diluted samples (50 μl) were added to the plate before the addition of the enzyme conjugate (100 μl) and a 60-min incubation at 37 °C. After washing, a substrate mixture (solutions A + B) was added and incubated for 15 min (37 °C, dark). Absorbance was read at 450 nm after stopping the reaction.

### Statistical Analysis

SPSS 23.0 (IBM; Armonk, NY, USA) was utilized to process data. A t-test and ANOVA were utilized to analyze differences between groups. The diagnostic value was assessed by the ROC curve. The chi-squared test was employed to analyze associations between the clinicopathological characteristics of PI and miR-144-3p level. Risk factors for PI were analyzed utilizing logistic regression. p < 0.05 was considered statistically significant.

## RESULTS

### Baseline Characteristics of the Two Groups

The PI group included 59 males and 55 females, with an average age of 44.10 ± 8.75 years, and the control group included 42 males and 56 females, with an average age of 42.30 ± 7.45 years. As demonstrated in Table 1, periodontal disease history (52.6% vs 27.6%), PLI (2.07 ± 0.83 vs 1.28 ± 0.45), SBI (2.86 ± 0.50 vs 1.53 ± 0.51), PD (5.01 ± 1.00 mm vs 3.01 ± 0.76 mm), FMPS (58.67 ± 8.41% vs 26.33 ± 6.32%), CAL (5.18 ± 1.47 mm vs 2.83 ± 0.72 mm), and the rate of bleeding on probing/suppuration (97.4% vs 17.3%) in the PI group were statistically significantly increased (p < 0.001). However, there was no significant difference in age, sex, smoking, or drinking between the two groups (p > 0.05).

**Table 1:** Baseline characteristics between the two groups

Variable	Control (n=98)	PI (n=114)	p-value
Age (years)	42.30 ± 7.45	44.10 ± 8.75	0.111
Sex (male/female)	42/56	59/55	0.196
Smoking (yes/no)	55/43	69/45	0.516
Drinking (yes/no)	44/54	57/57	0.458
Periodontal disease history (yes/no)	27/71	60/54	< 0.001***
PLI (scores)	1.28 ± 0.45	2.07 ± 0.83	< 0.001***
SBI (scores)	1.53 ± 0.51	2.86 ± 0.50	< 0.001***
PD (mm)	3.01 ± 0.76	5.01 ± 1.00	< 0.001***
FMPS (%)	26.33 ± 6.32	58.67 ± 8.41	< 0.001***
CAL (mm)	2.83 ± 0.72	5.18 ± 1.47	< 0.001***
Bleeding on probing/suppuration (yes/no)	17/81	111/3	< 0.001***
PI: peri-implantitis; PLI: plaque index; SBI: sulcus bleeding index; PD: probing depth; FMPS : full-mouth plaque scores; CAL: clinical attachment level; ***statistically significant at p < 0.001.			

### Effects of miR-144-3p in PI

The miR-144-3p level statistically significantly increased in PI (Fig 1a). miR-144-3p had a statistically significant diagnostic value in PI (AUC = 0.905, sensitivity = 82.46%, specificity = 86.67%) (Fig 1b). As shown in Table 2, periodontal disease history, PLI, SBI, PD, and miR-144-3p were risk factors for PI (p < 0.05). Because FMPS, CAL, and bleeding on probing/suppuration are major diagnostic criteria for PI, they were not included in the risk factor model to avoid circularity. Nonetheless, age, sex, smoking, or drinking were not risk factors for PI (p > 0.05).

**Fig 1:**
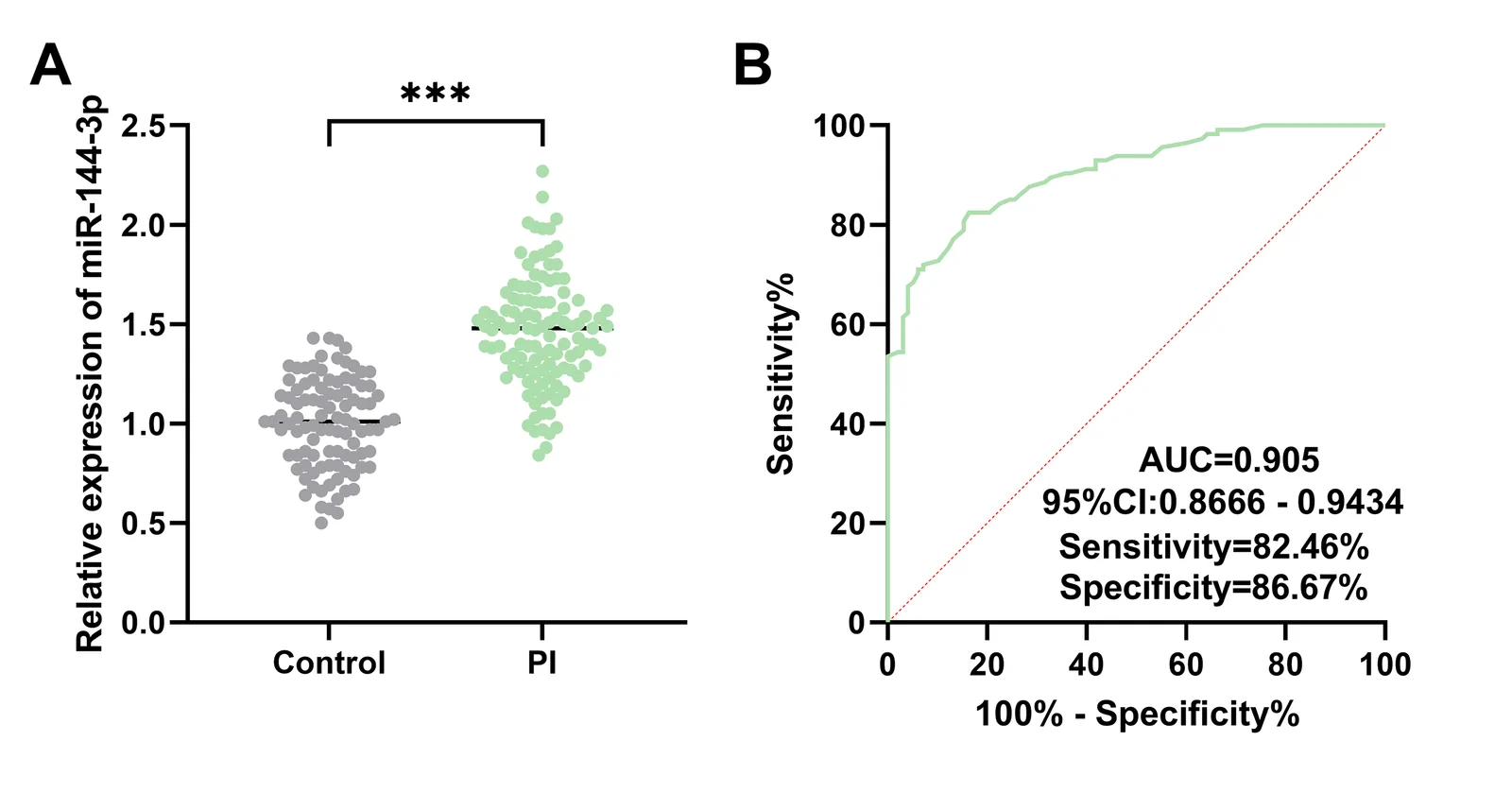
a. Relative expression of miR-144-3p in PI. b. The diagnostic value of miR-144-3p in PI. ***p < 0.001.

**Table 2:** Logistic regression analysis of risk factors for PI

Variable	OR	95 % CI for OR		p-value
		Lower	Upper	
Age	1.994	0.730	5.446	0.178
Sex	0.557	0.171	1.822	0.333
Smoking	0.594	0.214	1.654	0.319
Drinking	0.419	0.124	1.410	0.160
Periodontal disease history	3.587	1.160	11.088	0.027*
PLI	4.026	1.415	11.454	0.009**
SBI	6.483	2.098	20.034	0.001**
PD	4.321	1.369	13.637	0.013*
miR-144-3p	9.712	3.508	26.892	< 0.001***
PI: peri-implantitis; PLI: plaque index; SBI: sulcus bleeding index; PD: probing depth; *p < 0.05. ** p < 0.01.*** p < 0.001.				

### Correlation Between miR-144-3p Level and Baseline Characteristics of PI patients

PI patients were stratified into low and high miR-144-3p expression groups based on the median expression level. As demonstrated in Table 3, miR-144-3p level was closely related to periodontal disease history, PLI, SBI, PD, FMPS, and CAL (p < 0.05). Bleeding on probing/suppuration was observed in almost all PI patients, and three cases without this sign were all in the low miR-144-3p group, but the difference did not reach statistical significance (p = 0.064). Nevertheless, miR-144-3p level was not linked to age, sex, smoking, and drinking (p > 0.05).

**Table 3:** Association between clinicopathological features and miR-144-3p expression levels in PI

Variable	Total	miR-144-3p		p-value
	(n = 114)	Low (n = 54)	High (n = 60)	
**Age (years)**				
≤44	60	27	33	0.593
>44	54	27	27	
**Sex (male/female)**				
male	59	30	29	0.441
female	55	24	31	
**Smoking (yes/no)**				
yes	69	34	35	0.614
no	45	20	25	
**Drinking (yes/no)**				
yes	57	29	28	0.453
no	57	25	32	
**Periodontal disease history (yes/no)**				
yes	60	16	44	< 0.001***
no	54	38	16	
**PLI (scores)**				
≤2.07	55	40	15	< 0.001***
>2.07	59	14	45	
**SBI (scores)**				
≤2.86	60	36	24	0.004**
>2.86	54	18	36	
**PD (mm)**				
≤5.01	55	34	21	0.003**
>5.01	59	20	39	
**FMPS (%**)				
≤58.67	61	36	25	0.008**
>58.67	53	18	35	
**CAL (mm)**				0.016*
≤5.18	54	32	22	
>5.18	60	22	38	
**Bleeding on probing/suppuration (yes/no)**				0.064
yes	111	51	60	
no	3	3	0	
PI: peri-implantitis; PLI: plaque index; SBI: sulcus bleeding index; PD: probing depth; FMPS: full-mouth plaque scores; CAL: clinical attachment level;* p < 0.05. ** p < 0.01. *** p < 0.001.				

### Effect of miR-144-3p on PI Model

With the increase in LPS treatment time, the cell viability in HGFs decreased, while TNF-α and IL-1β increased (Figs 2a to 2c). Then, HGFs treated with 1 μg/ml LPS at 12 h were selected as the PI model. miR-144-3p was upregulated or downregulated in the PI model, which was confirmed (Fig 3a). In the PI model, overexpression of miR-144-3p reduced proliferation, while inhibition of miR-144-3p elevated proliferation (Fig 3b). In addition, overexpression of miR-144-3p elevated the levels of TNF-α and IL-1β, while inhibition of miR-144-3p reduced the levels of TNF-α and IL-1β (Figs 3c and 3d).

**Fig 2:**
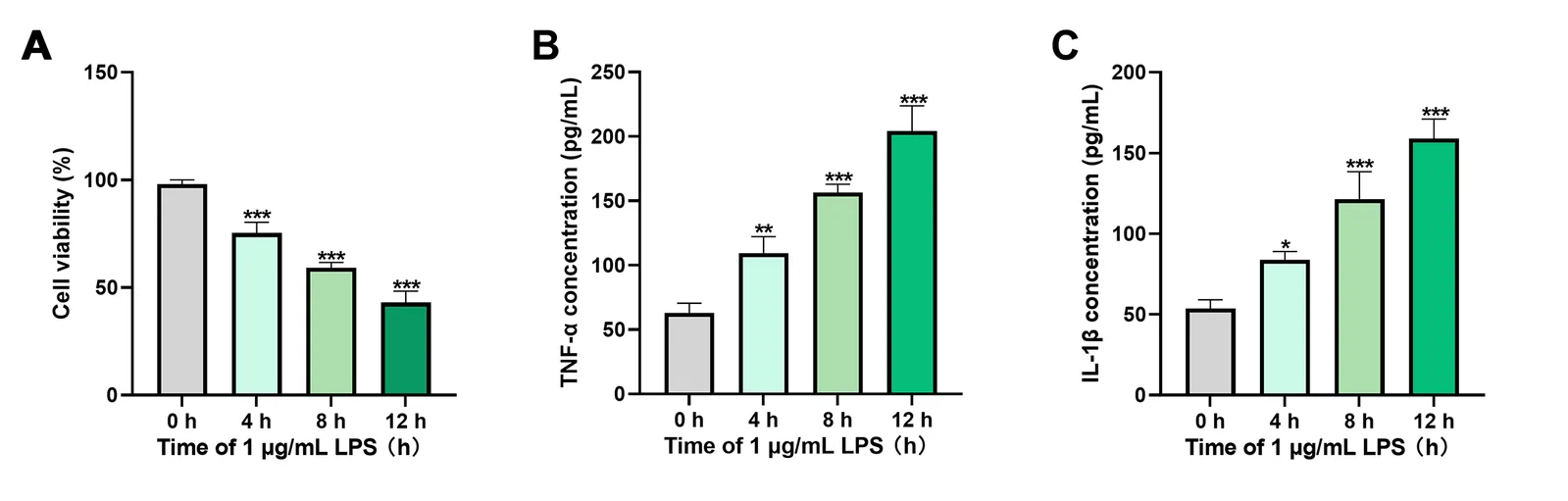
Human gingival fibroblasts (HGFs) were treated with 1 μg/ml of LPS for different durations. a. Cell viability in HGFs. b. The concentration of TNF-α. c. The concentration of IL-1β. *p < 0.05. ** p < 0.01. ***p < 0.001.

**Fig 3:**
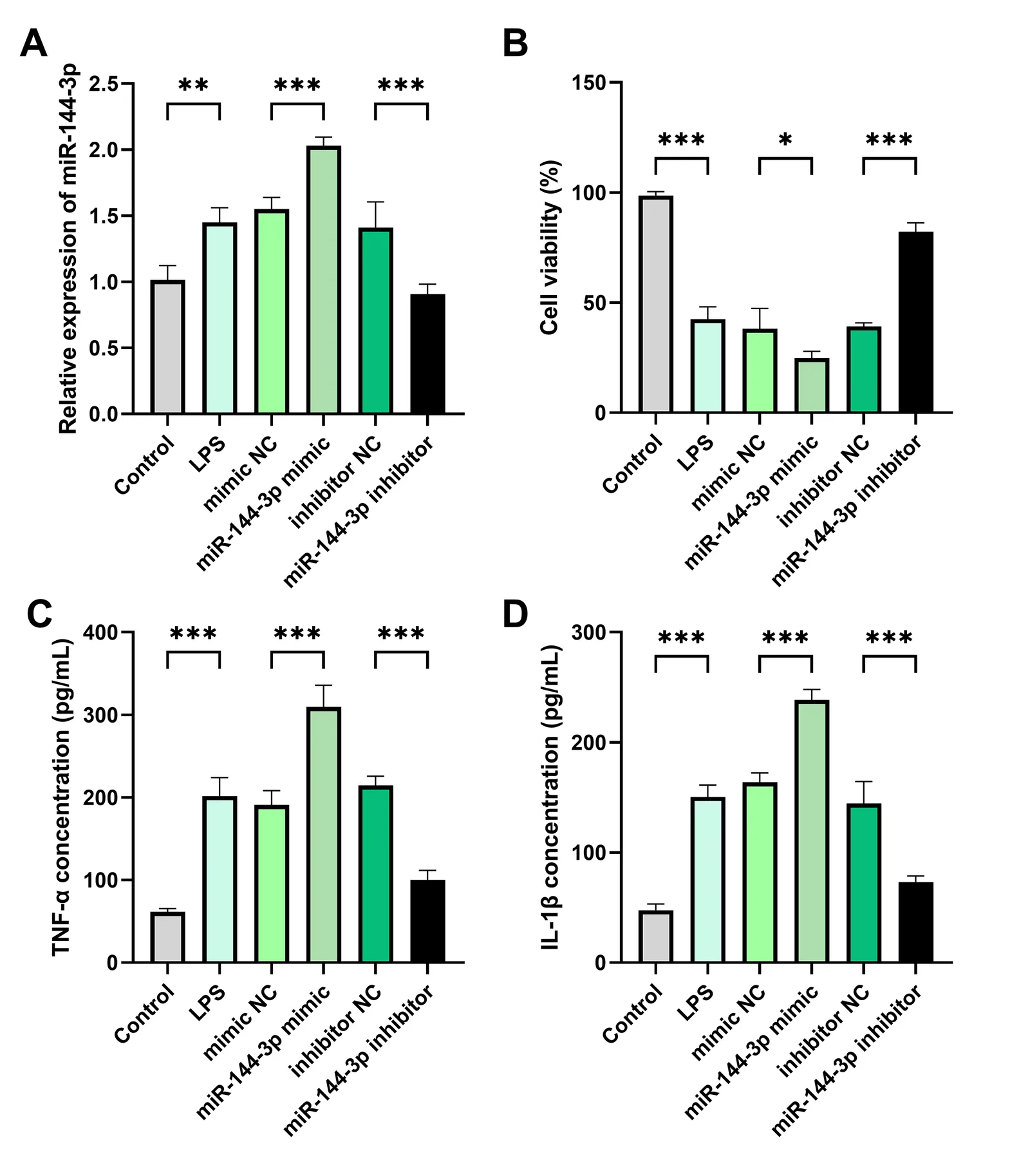
Effect of miR-144-3p on the PI model. a. Relative expression of miR-144-3p. b. Cell viability in the PI model. c. Concentration of TNF-α. d. Concentration of IL-1β. *p < 0.05. **p < 0.01. ***p < 0.001.

## DISCUSSION

Bacterial infections during or after dental implant surgery are the main cause of dental implant failure.^6^ PI is a pathological condition related to dental plaque that occurs in the tissues surrounding the implant.^28,30^ At present, the diagnosis of peri-implant diseases mainly relies on imaging and clinical parameters.^21^ PI exhibits clinical features similar to those of periodontitis, including obvious gingivitis, bleeding on probing, and bone loss on imaging,^5^ which were consistent with the baseline characteristics of PI patients in this study. The loss of implants usually has an impact on patients’ well-being (physical and and psychological), and at the same time brings huge economic losses. Early diagnosis of PI is crucial for understanding its pathological progression and prevention.^7^ Therefore, the current situation necessitates the development of new diagnostic biomarkers. Studies have shown that the functional role of miRNA makes it a potential therapeutic target for periodontal and peri-implant diseases.^1,20^ In this study, miR-144-3p had statistically significant diagnostic value in PI, indicating that it has the potential to serve as a diagnostic biomarker for PI. Furthermore, we found that miR-144-3p is a risk factor for PI.

The width of the keratinized mucosa around the implant is associated with reduced mucosal inflammation, decreased plaque accumulation, increased stability in the surrounding area, and prevention of mucosal atrophy, which can lead to implant loss.^25^ Periodontal indicators, including PLI, SBI, PD, FMPS, and CAL are commonly used to assess oral hygiene and periodontal inflammation, and traditional diagnoses relying on probing and radiographs have limitations.^11^ Our analysis revealed that miR-144-3p level was strongly linked with the key indicators of PI, suggesting that its expression was related to the severity of PI. Furthermore, as a non-invasive and objective biomarker, miR-144-3p exhibits high diagnostic accuracy (AUC = 0.905) and may serve as a complementary tool to conventional parameters, thereby enhancing the overall diagnostic performance.

The pathogenesis of PI has been shown to be mediated by miRNA.^42^ Among them, miR-144-3p participates in the development of chronic periodontitis. Since PI and periodontitis are both plaque-induced inflammatory diseases with similar pathological mechanisms, we speculated that miR-144-3p may also play a role in PI. To test this, HGFs treated with *P. gingivalis* LPS were selected as the PI model, because *P. gingivalis* is a key periodontal pathogen associated with plaque-driven inflammation.

In this model, we first examined the effect of miR-144-3p on cell proliferation. Previous studies have shown that miR-144-3p regulates proliferation in various cell types: in steroid-associated osteonecrosis, miR-144-3p reduces proliferation and osteogenic differentiation of BMSC.^32^ Notably, miR-144-3p also reduces proliferation in some tumors such as glioblastoma,^15^ multiple myeloma,^41^ and nephroblastoma.^17^ Similarly, we found that in the PI model, miR-144-3p inhibited proliferation.

Additionally, miR-144-3p also regulates inflammation in many diseases. In the mouse model of renal interstitial fibrosis, miR-144-3p promotes cellular inflammation.^36^ In Crohn’s disease, miR-144-3p serves as a biomarker for assessing mucosal inflammation.^9^ In mouse septic acute lung injury, miR-144-3p aggravates lung tissue destruction and inflammatory response.^35^ The above results are consistent with the trend of miR-144-3p’s effect on inflammatory factors in the PI model presented in our study. The above evidence indicates that miR-144-3p might participate in PI by regulating cell proliferation and inflammatory responses.

However, this study had some limitations. For instance, the number of samples available for analysis was limited. In addition, we only established a PI cell model and did not conduct in-vivo validation in mouse or rat models. In future research, animal PI models will be involved to further explore effects of miR-144-3p in PI.

## CONCLUSION

Overall, miR-144-3p increased in PI and had statistically significant diagnostic value in PI. miR-144-3p was validated as a risk factor for PI. In addition, the miR-144-3p level was closely related to pathological indicators of PI patients. In the PI model, miR-144-3p reduced proliferation and increased inflammatory factor levels, confirming that it has the possibility to become a biomarker of PI.

## REFERENCES
